# Metabolomic tool to identify soybean [*Glycine max* (L.) Merrill] germplasms with a high level of shade tolerance at the seedling stage

**DOI:** 10.1038/srep42478

**Published:** 2017-02-13

**Authors:** Jiang Liu, Baoyu Hu, Weiguo Liu, Wenting Qin, Haijun Wu, Jing Zhang, Caiqiong Yang, Juncai Deng, Kai Shu, Junbo Du, Feng Yang, Taiwen Yong, Xiaochun Wang, Wenyu Yang

**Affiliations:** 1 Key Laboratory of Crop Ecophysiology and Farming System in Southwest, Ministry of Agriculture, Chengdu 611130, China; 2 Institute of Ecological Agriculture, Sichuan Agricultural University, Chengdu 611130, China

## Abstract

The isoflavone profiles of seeds of various soybean genotypes with different levels of shade tolerance at the seedling stage were investigated. High-performance liquid chromatography (HPLC) was used to quantify 12 isoflavones, and the data were analyzed using a multivariate statistical analysis. Combined with field experimental data and an orthogonal partial least-squares discriminant analysis (OPLS-DA), several aglycones (genistein (GE), daidzein (DE), and glycitein (GLE)) were selected and identified as key compounds involved in the shade tolerance of soybean seedlings. Additional correlation analysis and laboratory shading stress experiments with soybean seedlings also confirmed the function of these selected isoflavones, especially GE, in the discrimination of soybean seedlings with different levels of shade tolerance. Furthermore, the structure–antioxidant activity relationships between a range of isoflavones and the plant shade-tolerance mechanism are discussed. Targeted metabolomic analyses of isoflavones could reveal the diversity of shade tolerance in soybean seedlings, thus contributing to the breeding of excellent soybean varieties.

Multi-species cropping is an ecological planting pattern that provides a more favorable environment for enhancing the land utilization ratio, especially in developing countries. There are several benefits of intercropping systems, such as minimizing weed competition^1^,^2^, reducing insect^3^ and disease incidence^4^, and increasing nitrogen uptake^5^. These benefits can ultimately improve total intercropped yields. Maize-soybean intercropping plays a particularly important role in agricultural production in the southwestern regions of China^6^. However, there are several disadvantages associated with this type of intercropping system. Previous research demonstrated that under this type of intercropping system, the microclimate environment within the soybean crop canopies was changed, particularly with respect to the light intensity and spectral properties of the canopies. The ratio of red light (655 nm to 665 nm) to far-red light (725 nm to735 nm) (R/FR) was 1.05–1.35 in sole planting with soybean, whereas the R/FR values ranged from 0.55 to 0.85 in the maize-soybean relay strip intercropping system^7^. In a maize-soybean relay strip intercropping system, soybean grows in the shade environment of the maize canopy. As shade stress increases, soybean photosynthetic efficiency, specific leaf weight, and chlorophyll a/b ratio decline^8^. The poor light environment in an intercropping system reduces the number of seeds per plant, the seed weight per plant, the mass of aerial products per plant, and the 100-seed weight, ultimately resulting in an observable decrease in the yield of intercropped soybean^9^. Additionally, stalk lodging and vining of soybean during the co-growth stage also reduces the quality and yield of intercropped soybean^10^.

There are two main strategies to address the problems arising from the effect of shade in intercropping systems: field arrangement and variety selection. Previous research indicated that the light intensity and spectral properties of the canopies varied with row spacing in a maize-soybean relay strip intercropping system^11^. Appropriate field arrangement could be used to achieve high yields in maize-soybean intercropping. Under optimum bandwidth and narrow-row spacing in maize, total intercrop yields were higher than the sole-cropped maize and soybean, and the land equivalent ratios of the intercropping systems were higher than 1.3^12^.

Another main strategy is the selection of suitable varieties, which consists of two sub-components: compact maize and shade-tolerant soybean. On one hand, maize varieties with converged or semi-compact leaves may be selected. Previous research revealed that the combination of compact maize with the appropriate distance between maize and soybean rows improved the photosynthetically active radiation (PAR) intensity and transmittance in maize-soybean relay strip intercropping systems. Regulating the light associated with the maize plants can lead to optimization of the distribution of PAR intensity and can improve the photosynthetic characteristics of intercropped soybean^13^. However, shade-tolerant soybean germplasm resources can be used as a direct and efficient approach to relieve the effect of shade resulting from the maize canopy. In fact, evaluation methods have been developed that were successfully used to screen several types of soybean germplasm for good shade tolerance. The current evaluation methods depend mainly on agronomic characteristics in sole and intercropped systems, such as vining severity index and weighted vining index^10^. Although these evaluation methods are relatively useful, the process is too cumbersome for rapid and convenient evaluation. Generally, field evaluation experiments take more than two years and are vulnerable to the external environment, leading to inaccurate results^14^. Therefore, research is warranted to establish a simple, rapid, and convenient method for the evaluation and screening of shade-tolerant soybean germplasms.

Soybean seeds contain high concentrations of isoflavones as health-promoting factors, which also play important roles in the ontogenic tolerance to various biotic or abiotic stresses. Previous findings indicate that isoflavonoid metabolism plays an important role in plant stress resistance responses. However, it is not clear whether there is a positive relationship between the isoflavonoid profile and shade tolerance in soybeans. Our preliminary experiment analyzed isoflavone diversity in 144 soybean germplasms collected from different regions of southern China (results to be published elsewhere). This study demonstrated that genetic factors play a more important role in isoflavone biosynthesis than geographical environmental factors. Vast phytochemical diversity, especially in the most variable aglycones in soybean seeds among different soybean germplasms, was definitively decided by genetic factors. Consequently, we hypothesized that there is a specific isoflavonoid profile, especially as observed in aglycone variation, that may be used as a marker to characterize tolerant germplasms. Thus, the present study was performed to evaluate the role of isoflavones in various soybean genotypes with different shade tolerance at the seedling stage. Additionally, the orthogonal partial least-squares discriminant analysis (OPLS-DA) technique was used to obtain robust and clear classification models and to identify the key compounds involved in the shade tolerance of soybean seedlings^15^.

## Results

### Shade Tolerance and Isoflavone Profiles of the Soybean Germplasm

Weighted index of sensitivity (WIS) values were calculated based on various agronomic characteristics according to the sensitivity of the soybean seedlings to shade generated by the intercropped maize. The WIS values represent the shade tolerance of the soybean germplasm. As shown in Table 1, the tested soybean germplasms could be divided into three main groups: 1. shade-tolerant soybean germplasms (ND12 and 14011) with WIS values < 0.6, indicating no sensitivity to shade stress; 2. moderately shade-tolerant soybean germplasms (14022, 14015, 14027, and 14057) with WIS values of approximately 1.0; and 3. susceptible soybean germplasms (14059, 14055, and C103) with WIS values > 1.2, indicating sensitivity to shade stress.

The concentrations of twelve isoflavones, including daidzin (DG), glycitin (GLG), genistin (GEG), malonyldaidzin (MD), malonylglycitin (MGL), acetyldaidzin (AD), acetylglycitin (AGL), malonylgenistin (MG), daidzein (DE), acetylgenistin (AG), glycitein (GLE), and genistein (GE), in the above nine soybean germplasm types were determined using HPLC. The concentrations of various types of isoflavones were calculated using the following equations: total aglycone (T-e = GE + DE + GLE), total glucoside (T-g = GEG + DG + GLG), total malonylglucoside (T-m = MG + MD + MGL), total acetylglucoside (T-a = AG + AD + AGL), total G-type isoflavonoids (To-G = GE + GEG + AG + MG), total D-type isoflavonoids (To-D = DE + DG + AD + MD), and total GL-type isoflavonoids (To-GL = GLE + GLG + AGL + MGL). Malonylglucosides and β-glucoside were the most prevalent (>85%) isoflavonoids observed in soybean seeds (Supplemental content – Table S1). According to the chemical skeleton of the soy isoflavonoids, G-type and D-type isoflavonoids were predominant in the soybean seeds (Supplemental content – Table S1). The soybean isoflavone concentrations varied widely among soybean germplasm types. For example, the coefficient of variation (CV) of T-g and T-e reached 22.47% and 25.62%, respectively, with glucoside and aglycone as the main sources of variation. Furthermore, the average concentration of isoflavones in the soybean seeds was approximately 1.81 mg/g. The soybean seeds of germplasms C103, 14055, and ND12 yielded the highest concentrations (2.295, 2.291, and 1.992 mg/g, respectively) (Supplemental content – Table S1).

### Multivariate OPLS-DA Analyses of Isoflavones

**Figure 1:**
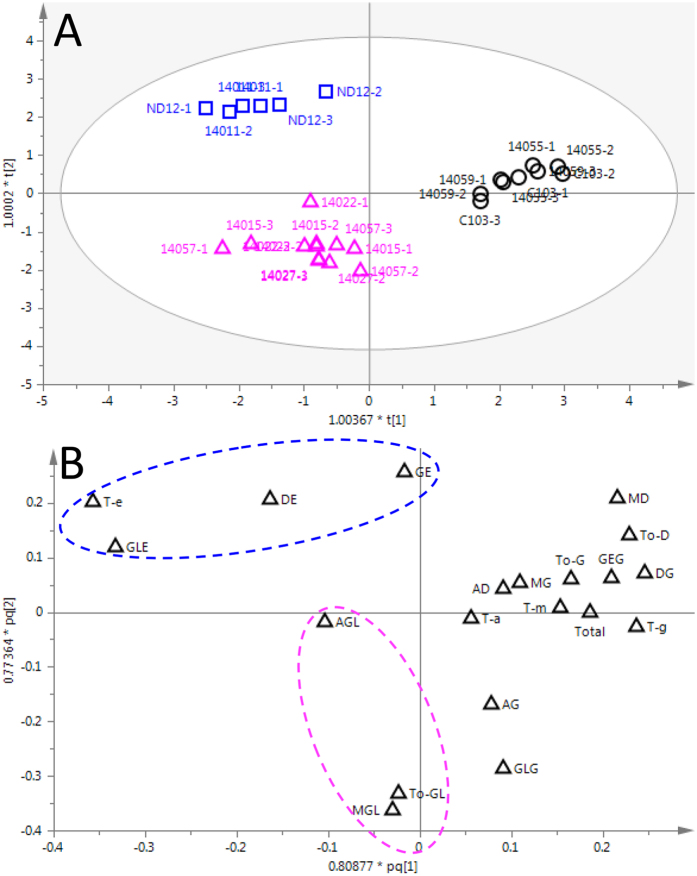
OPLS-DA score plot (**A**) and corresponding loading scatter plot (**B**) of soybean seeds with different levels of shade tolerance. The blue box, red triangle, and black circle represent the shade-tolerant, moderately shade-tolerant, and susceptible samples, respectively.

To investigate the relationship between the isoflavone profile of the soybean seeds and their shade-tolerant phenotype at the seedling stage, the isoflavone concentrations of nine soybean genotype seeds exhibiting three different levels of shade tolerance were measured, and a multivariate statistical analysis was applied (Table 1). Specifically, a supervised multivariate model (OPLS-DA) was constructed based on the shade-tolerance characteristics. The parameters of the cross-validation plot, i.e., *R*^2^(X) = 0.977, *R*^2^(Y) = 0.926, and *Q*^2^ = 0.845, demonstrated that the model was credible. Three soybean groups were separated in the OPLS-DA score plot: susceptible soybeans were separated from shade-tolerant and moderately shade-tolerant soybeans based on the first OPLS principal component (Fig. 1A). The shade-tolerant and moderately shade-tolerant soybeans were separated along the second OPLS principal component. The corresponding loadings scatter plot, presented above the OPLS-DA model (Fig. 1B), indicates that the contents of several metabolites were higher in the shade-tolerant soybeans, e.g., GE, DE, GLE, and their total content, T-e. Interestingly, all the isoflavones were aglycones. Although the total concentration of isoflavones was slightly higher in C103 (2.295 mg/g) than in ND12 (1.992 mg/g), the relative level of aglycones among the total content of isoflavones was significantly higher in ND12 (5.72%) than in C103 (1.95%) (Supplemental content – Table S1). The percentage concentrations of GE, DE, GLE, and T-e were all highest in germplasms 14011 and ND12, which, as the most prominent shade-tolerant soybeans, had the lowest WIS values (Table 1). Additionally, GL-type isoflavones, including AGL, MGL, and To-GL, were highlighted from the second OPLS principal component, which further distinguished shade-tolerant and susceptible soybeans. AGL, MGL, and To-GL contributed the most to the separation of these two soybean groups (Fig. 1B). These results indicate a certain function of GL-type isoflavones in the differentiation of soybean germplasms. Based on the information from the above OPLS-DA model and quantitative analysis, aglycones play a key role in the distinction of soybeans with different levels of shade tolerance.

### Correlation Analysis Between Isoflavone Contents and WIS

**Figure 2:**
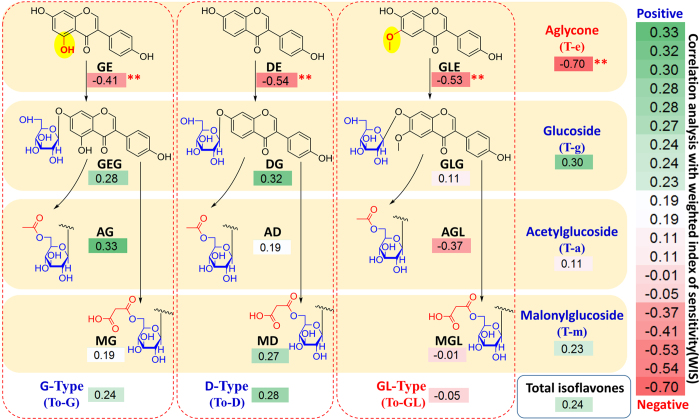
Correlation analysis between isoflavone contents and the weighted index of sensitivity (WIS). The numbers in the colored boxes are correlation coefficients, which are expressed according to the color scale shown beside the image. **Indicates significance at the *P* < 0.01 level, and *indicates significance at the *P* < 0.05 level.

To further investigate the relationship between the chemical structures of the isoflavones in the soybean seeds and their corresponding shade-tolerant phenotypes at the seedling stage, correlation analysis between the isoflavone contents and WIS values was conducted. Figure 2 clearly indicates that the concentrations of the aglycones (GE, DE, GLE, and T-e) and GL-type isoflavonoids (GLE, AGL, MGL, and To-GL) were negatively correlated with the WIS, whereas the other isoflavones were positively correlated with the WIS value. In addition, significant negative correlations were observed between the concentrations of aglycones (GE, DE, GLE, and T-e) and the WIS at the 0.01 significance level. Although there was no significant correlation between the total isoflavone content and the WIS, the highest negative correlation coefficient was found for the total aglycone content (T-e). Overall, the aglycone content in soybean seeds was statistically significantly correlated with shade tolerance at the seedling stage. These results suggest that aglycones are potential metabolite biomarkers in shade-tolerant soybeans.

### Comparison of Isoflavonoid Biosynthetic Responses to Shade Signals between Typical Germplasms

**Figure 3:**
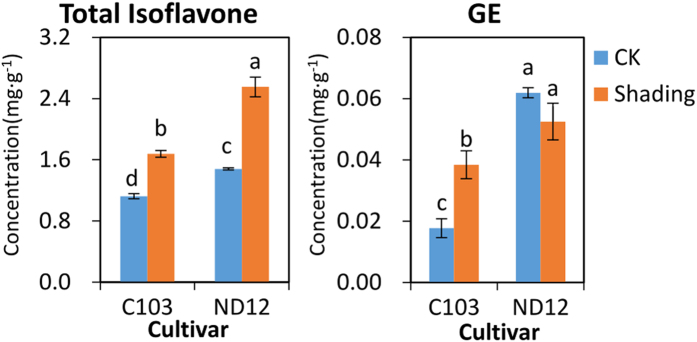
Concentrations of total isoflavones and genistein (GE) in the leaves of soybean seedlings grown under normal and shading conditions.

To further elucidate the relationship between isoflavone content and soybean seedling shade tolerance, a laboratory experiment was conducted in a phytotron chamber. The differential shade responses of the isoflavonoid profiles were compared between a shade-tolerant soybean germplasm ND12 (WIS = 0.51) and susceptible soybean germplasm C103 (WIS = 1.26) that are widely cultivated throughout southwestern China. Under the controlled environment, shade treatment induced variations in the isoflavonoid profiles in soybean leaves that differed between these two typical varieties. As shown in Fig. 3, the total isoflavonoid content increased significantly from 1.123 mg/g to 1.676 mg/g in the leaves of C103 and from 1.480 mg/g to 2.551 mg/g in the leaves of ND12; thus, the size of the increase was greater in ND12 (Supplemental content – Table S2). Only one of the isoflavonoid aglycones, GE, was detected in the tested soybean leaves and increased significantly from 0.018 mg/g to 0.038 mg/g in C103. Although there was no significant change in GE content in ND12 after shading treatment (from 0.062 mg/g to 0.052 mg/g), the concentration was significantly higher than in any sample of C103. Although the total concentration of isoflavones in C103 seeds (2.295 mg/g) was higher than that in ND12 (1.992 mg/g), the proportion of aglycones in the total isoflavone content was significantly higher in ND12 leaves (5.72%) than that in C103 (1.95%) (Supplemental content – Table S2). Taken together, the aglycone content in soybean seedling leaves was significantly higher in the germplasm with a high level of shade tolerance at the seedling stage, especially under shading treatment. These results suggest that aglycones, especially GE, play an important role in shading stress tolerance responses.

## Discussion

### Targeted Metabolomic Analysis of Isoflavones Reveals Shade-Tolerance Diversity of Soybean Seedlings

Shade-tolerant soybean selection can be used as a direct and efficient approach to reduce the influence of shade from the maize canopy. The current evaluation method was conducted based on a real field experiment, which can take a long time^14^, especially when testing numerous germplasm types. Presently, the development of a convenient evaluation method for the preliminary screening of shade-tolerant soybean germplasms is urgently required. OPLS is a useful multivariate projection method that extracts linear relationships from data blocks by removing structured noise^16^. Compared with principal component analysis (PCA), hierarchical cluster analysis (HCA), and regular partial least squares (PLS) regression, OPLS provides a simpler and more effective solution to obtain robust and clear classification models and to identify relationships between the predictive or uncorrelated latent space underlying these models^17^. In our established OPLS-DA model, the parameters of the cross-validation plot, especially *R*^2^(X) and *R*^2^(Y), approached 1.0, and all the soybean groups were clearly separated. It was demonstrated that the fitting accuracy of these models was very high, and the models were credible. Additionally, the results of the correlation analysis also confirmed the function of the selected isoflavones in the discrimination of soybean seedlings with various levels of shade tolerance. In conclusion, this isoflavone profiling analysis in combination with the OPLS-DA model allowed us to predict the seedling shade tolerance potential of numerous soybean germplasm types without conducting tedious field experiments. For example, to screen out soybean varieties with higher shade tolerance from a major community of soybean germplasms (i.e., more than 500), initial quantitative analysis of isoflavones by HPLC can be followed by multivariable statistical analysis (i.e., PCA or HCA) of the data to determine their isoflavone profiles. Soybean germplasms may be divided into several groups, and the groups with a higher concentration ratio of aglycones, especially GE, can be selected as candidates with seedling shade tolerance for further confirmation.

### Isoflavonoid Metabolism is Closely Related to Shade Tolerance of Soybean at the Seedling Stage

Isoflavone biosynthesis and accumulation in soybean is determined by genotypic and environmental effects as well as environment × genotype (G × E) interactions. Multiple environmental factors (e.g., temperature, UV radiation, and nutrients in soil) have been regarded as important drivers of variability in flavonoids^18^. Environmental factors have a large impact on isoflavone metabolism in the soybean plant, and the variation of the isoflavone profile *in planta* also plays an important role in ontogenic tolerance to various biotic or abiotic stresses. Previous studies indicated that soy isoflavones appear to be important in fungitoxicity and insect resistance of cultivated soybean and other plants in production^19^,^20^. Other studies have shown that soy isoflavone also has benefits for enhancing drought^21^ and salt tolerance^22^ and protection from UV-B-induced DNA damage^23^. Furthermore, soy isoflavones appear to play an important function in light stress and antioxidant defense^24^,^25^. Acclimation mechanisms such as alteration of leaf chemistry, especially UV-B-absorbing flavonoids, govern the overall impact of UV-B on plants. Inherent or induced flavonoids in plant can protect the photosynthetic processes even under extreme light conditions^26^. In our current research, isoflavone aglycones, especially GE, which are major antioxidative components^27^, were highlighted based on multivariate statistical analysis and shading stress simulation experiments. The increased amount of such aglycones under shading treatment could be an acclimation mechanism adapted by soybean seedlings to minimize shading-induced damage.

### Structure–Antioxidant Activity Relationships as a Mechanism of Shade Tolerance in Soybean Seedlings

Many studies have indicated that reactive oxygen species are produced under various stress conditions, including photoinhibition, atmospheric pollutants, waterlogging, drought, and pathogens^28^. Oxidative stress is an important phenomenon in many biological systems. Under these oxidative stresses, the active oxygen balance is broken, and the accumulation of reactive oxygen species produces a toxic effect on the cell membrane, leading to apoptosis. Based on our previous studies, many parameters associated with peroxidation were detected in intercropped soybean subjected to shading by maize^29^. The application of certain chemical control substances could increase superoxide dismutase activity, peroxidase activity, and proline content while decreasing the malondialdehyde content and lipid peroxidation, thus protecting the cell membrane integrity and organic function of intercropped soybean^29^. Considerable literature has showed that numerous antioxidases and compounds are purified as natural antioxidants in plants, and they have been identified as essential components of the defense mechanism^28^. There are many types of natural products, including phenolic acids, flavonoids, alkaloids, and polysaccharides, that exhibit the ability to react with free radicals, thus reducing oxidative damage^30^,^31^. In particular, flavonoids are a large group of secondary plant polyphenolic compounds with significant antioxidant and chelating properties^32^,^33^. Although there is considerable debate about the mechanism of the protective action of flavonoids^34^, it is known that the antioxidant abilities are determined by the chemical structure of the antioxidant, and the presence of hydroxyl substituents in the flavonoid nucleus enhances antioxidant activity^35^.

Soybean isoflavone is a natural compound with high antioxidant activity^36^. Soybean seeds contain twelve typical isoflavones (Fig. 2) that are present in four different forms: aglycones, *β*-glucosides, malonylglucosides and acetylglucosides. The number and position of hydroxyl groups largely determine the radical-scavenging activity of flavonoids^37^. *ortho*-Dihydroxy groups are the most important structural feature for high activity for flavonoids^38^. The glycosylation and methoxylation of flavonoids could decrease their inhibitory effects^32^ due primarily to steric effects of the bulky substituent, which leads to a reduction in the inhibitory efficiency of the nearby hydroxyl groups^39^. In our current research, all aglycones, including DE, GLE, GE, and T-e, were selected using multivariate statistics and appeared to contribute to soybean shade tolerance (Fig. 1). Blocking the active hydroxyl function by glycosylation caused a decrease in inhibitory effects (Fig. 2, aglycones vs. glucosides). GE was identified in the shade stress simulation experiment as the antioxidant with the highest activity, due to its two active hydroxyl groups^38^. Additionally, the methoxy group (methoxylation, electron donation) might play a role in increasing isoflavonoid activity (Fig. 2, GL-type vs. D-type and G-type)^40^ by increasing their lipophilicity^41^ and membrane partitioning^32^.

In conclusion, the above-mentioned current and previous studies indicate that soy isoflavones, especially aglycone, are the key secondary metabolites that correlate with shade tolerance in soybean seedlings. These individual metabolites may provide additional insight into the shade tolerance and adaptation of legume plants. The metabolomic analysis based on the OPLS-DA model to predict the seedling shade tolerance potential of soybean germplasm types was verified.

## Methods

### Plant Materials and Experimental Design

Nine soybean genotypes (ND12, 14011, 14022, 14015, 14027, 14057, 14059, 14055 and C103), which were collected from different sites in the Sichuan Province of China in 2010, were used in this study (Table 1). The genotypes Nandou No. 12 (ND12) and C103 (Nan032-4) are conventional cultivars with high and low shade tolerance in southwestern China. All soybean genotypes were grown in the experimental field of the Sichuan Agricultural University (SICAU) at Ya’an in China (29°59′N, 103°00′E) from 2011 to 2014. The laboratory experiments were conducted at the research center of SICAU in 2016.

#### Experiment 1

**Figure 4:**
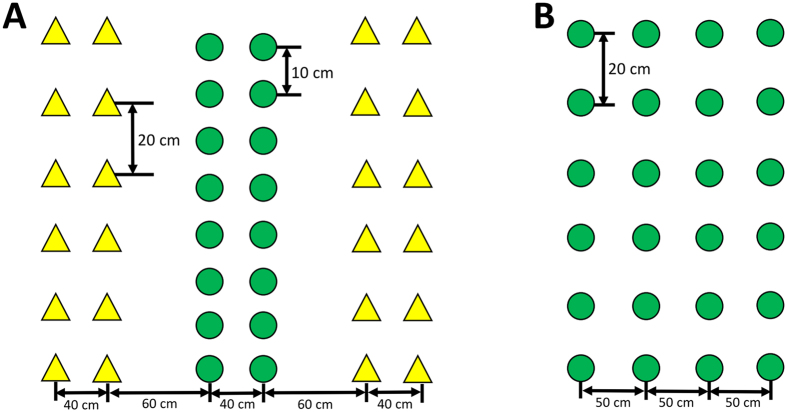
Planting patterns of relay strip intercropping (**A**) and sole cropping (**B**). The yellow triangles and green circles represent maize and soybean, respectively.

The germplasm evaluation experiment was performed from 2013 to 2014 after two years of acclimation. The field experimental design consisted of a two-factor randomized block design with three replicates. The two factors—nine soybean varieties and planting patterns (sole cropping and maize-soybean intercropping)—were analyzed for various agronomic characteristics based on our previous method with some modifications^42^. All tested soybean germplasms were intercropped with maize (*Zea. mays* L. cv. Chuandan No. 418), and the maize planting patterns that were used are shown in Fig. 4A. In the intercropping treatments, the ratio of maize to soybean rows was 2:2, and a single strip of each row had the same width (200 cm). Soybeans were planted in wide rows before the reproductive stage of maize with a row spacing of 40 cm and plant spacing of 10 cm. The maize-soybean intercropping pattern was 160 + 40 cm wide-narrow row spacing. The PAR transmittance of the soybean canopy was 47.5%, which was measured using LI-191SA quantum sensors (LI-COR Inc., Lincoln, NE, USA). In the sole cropping system, all soybean germplasms were planted in solid rows with 50 cm row spacing and 20 cm plant spacing (Fig. 4B). The PAR transmittance of the soybean canopy was 100%^43^. Each plot measured 6 × 7 m. Maize was sown on 26 March and harvested on 5 August. Soybean was sown on 13 June, when the maize plants were at the V12 stage, and had a 53-day coexistence period. Ten plants were randomly selected for each genotype in each replication, and those plants were averaged to provide one number per replication.

#### Experiment 2

The shade response comparison experiment for soybean isoflavonoids was conducted in the phytotron chamber of SICAU in 2016. The plants were germinated in a thermostat-controlled chamber for 2 days, and the seedlings were then transferred into standard Hoagland’s nutrient solution^44^. After 3 days, the seedlings in the cotyledon stage (Vc) were transferred into growth chambers (model LED-41L2; Percival Scientific Inc., Perry, IA). FR and R light was supplied using light-emitting diode light sources, with 7 days of shading and control (CK) treatments. A photoperiod of 12/12 h (light/dark), temperature of 25/15 °C (day/night), carbon dioxide concentration of 350 μmol/mol, and air relative humidity of 60% were maintained throughout the experiment. The laboratory experimental design consisted of a two-factor randomized block design with three replicates. Two typical varieties, ND12 (shade-tolerant soybean germplasm) and C103 (susceptible soybean germplasm), which are widely cultivated throughout southwestern China at present, were used as plant materials in the experiment. The second factor was two light treatments: shading (light intensity: 210 mol·m^2^·s^−1^, R/FR = 0.35) and CK (light intensity: 440 mol·m^2^·s^−1^, R/FR = 1.15). Three replicates were performed for each treatment. The treatment samples were harvested in the first trifoliate leaf stage (V1). The top first trifoliate leaves of the treatment and control plants were collected, immediately frozen in liquid nitrogen, and stored in air-tight tubes at −80 °C until further analysis.

#### Shade Tolerance Evaluation at the Seedling Stage

The shade tolerance evaluation was conducted according to the weighted index of sensitivity (WIS) in response to shade from the intercropped maize. The WIS value was integrated and calculated using previously published methods with certain modifications^10^,^45^. Agronomic soybean characteristics (main stem length, average internode length, hypocotyl length, node number, stem diameter, and breaking strength) were measured when grown in the maize-soybean intercropping (IT) system and the soybean sole cropping (SC) system. Breaking strength was measured on the 4^th^ internode using a digital force tester (YYD-1, Top Instrument, Zhejiang, China) and presented as snapping resistance (newtons, N). All the data were measured at the beginning of the blooming stage of the soybeans. A higher WIS value indicates that soybean seedlings are more sensitive to intercropping shade; it also indicates the weak shade resistance of a particular soybean genotype. The WIS can be expressed as follows (Eq. 2):


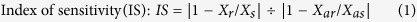


where X_r_ is the value of the agronomic characteristic of the single variety measured in the IT system; X_s_ is the corresponding value in the SC system; X_ar_ is the average value of the agronomic characteristics of all tested varieties in the IT system; and X_as_ is the corresponding value in the SC system.


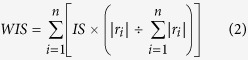


where r_*i*_ is the correlation coefficient between the IS of a single agronomic characteristic and the average IS value of all agronomic characteristics. 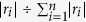 is the index weight, which represents the importance degree of the number *i* index.

#### Isoflavone Identification and Quantification

Isoflavone extraction, identification, and quantification were performed using an Agilent 1100-series HPLC system equipped with an ultraviolet spectroscopy detector based on our previously published methods with certain modifications^43^. Briefly, approximately 100.00 mg of each replicate was transferred into a pre-cooled reaction tube, and 5 mL of a pre-chilled MeOH/H_2_O (80/20 v:v) extraction solution was then added. The samples were vortexed for 10 s and extracted at room temperature for 3 h in an ultrasonic water bath (40 KHz). The samples were centrifuged at 11,000 g and 4 °C for 10 min, and the supernatant fluid was then filtered through a syringe filter (0.22 μm) and injected directly into the HPLC system. The main chromatographic conditions were as follows: mobile phase consisted of eluent A (0.1% acetate solution, v/v) and eluent B (acetonitrile) at a flow rate of 0.8 mL·min^**−1**^ and a column temperature of 30 °C; the UV detector was set to 260 nm; linear gradient: 85–80% of eluent A (0–30 min), 80–60% of eluent A (30–60 min), and 60% eluent A (60–70 min); the injection volume was 5 μL. Twelve isoflavones, including DG, GLG, GEG, MD, MGL, AD, AGL, MG, DE, AG, GLE, and GE, were quantified in absolute terms via linear regression of their corresponding standards (Supplemental content – Table S3). Identification of the isoflavones was achieved by comparing sample retention times with the standard compounds (Supplemental content – Fig. S1). Deionized water and HPLC-grade solvents (Fisher, Hampton, NH, USA) were used to prepare the solutions. The twelve isoflavone standard compounds were purchased from Wako Pure Chemical Industries Co., Ltd. (Japan).

#### Metabolomic Analysis

OPLS-DA was employed to summarize the systematic alteration of samples using SIMCA-P13.0 software (Umetrics, MKS Instruments Inc., Umea, Sweden). All isoflavone concentration data were visualized by plotting the principal component scores in which each coordinate represents an individual biological sample. Based on the OPLS-DA loadings scatter plot, maximum information was extracted from the data set to isolate the metabolites responsible for differences among groups. After multivariate statistical analysis, the significance of each metabolite in the group discrimination was further subjected to correlation analysis to construct mathematical models consisting of various isoflavone contents and the WIS; SPSS (version 20.0; SPSS, Chicago, USA) was used for model construction. Duncan’s multi-range test was used when the samples exhibited significant differences at the *P* < 0.05 level.

## Additional Information

**How to cite this article**: Liu, J. *et al*. Metabolomic tool to identify soybean [*Glycine max* (L.) Merrill] germplasms with a high level of shade tolerance at the seedling stage. *Sci. Rep.* **7**, 42478; doi: 10.1038/srep42478 (2017).

**Publisher's note:** Springer Nature remains neutral with regard to jurisdictional claims in published maps and institutional affiliations.

## Supplementary Material

Supplementary Information

## Figures and Tables

**Table 1:** Material Information.

Code	Name	Origin	Weighted index of sensitivity (WIS)	Note
ND12	NanDou No. 12	Nanchong, Sichuan	0.51	cultivar
14011	LUOSHI	Xichang, Sichuan	0.52	wild
14022	Gongxuan No. 5	Zigong, Sichuan	0.97	cultivar
14015	HUI	Xichang, Sichuan	1.02	wild
14027	MAO	Jiuzaigou, Sichuan	1.06	wild
14057	BAYUE	Pingchang, Sichuan	1.09	wild
C103	Nan032-4	Nanchong, Sichuan	1.26	cultivar
14059	DONG	Yibing, Sichuan	1.80	wild
14055	YINGSHAN	Muchuan, Sichuan	1.82	wild
