# Supplementary material for: Metabolomic tool to identify soybean [Glycine max (L.) Merrill] germplasms with a high level of shade tolerance at the seedling stage
Source: Sci Rep. 2017 Feb 13;7:42478. doi: 10.1038/srep42478 (PMC5304147; doi:10.1038/srep42478)
Supplement: Supplementary Information [file srep42478-s1.doc]

**Metabolomic tool** **to identify soybean [*Glycine max* (L.) Merrill] germplasms with** **a high level of shade tolerance at the** **seedling stage**

Jiang Liu^1,2,a*^, Baoyu Hu^1,a^, Weiguo Liu^1,2,a^, Wenting Qin^1,a^, Haijun Wu^1^, Jing Zhang^1^, Caiqiong Yang^1^, Juncai Deng^1^, Kai Shu^1,2^, Junbo Du^1,2^, Feng Yang^1^, Taiwen Yong^1^, Xiaochun Wang^1^, and Wenyu Yang^1^,^*^

**Supplementary content–****Table S1.** Variation in isoflavonoids in various types of soybean germplasm

| Sample | Isoflavone contents (mg/g) | | | | | | | | | | | |
| --- | --- | --- | --- | --- | --- | --- | --- | --- | --- | --- | --- | --- |
|  | DG | GLG | GEG | MD | MGL | MG | AD | AGL | AG | DE | GLE | GE |
| ND12 | 0.465±0.046 | 0.046±0.025 | 0.468±0.030 | 0.410±0.031 | 0.044±0.057 | 0.366±0.035 | 0.049±0.051 | 0.022±0.044 | 0.006±0.116 | 0.029±0.048 | 0.061±0.121 | 0.024±0.026 |
| 14011 | 0.208±0.015 | 0.102±0.013 | 0.320±0.007 | 0.215±0.015 | 0.089±0.065 | 0.249±0.018 | 0.036±0.027 | 0.021±0.029 | 0.006±0.072 | 0.019±0.020 | 0.058±0.061 | 0.019±0.017 |
| 14022 | 0.328±0.022 | 0.171±0.017 | 0.418±0.024 | 0.295±0.022 | 0.156±0.008 | 0.304±0.041 | 0.045±0.027 | 0.020±0.093 | 0.010±0.018 | 0.021±0.026 | 0.039±0.049 | 0.021±0.017 |
| 14015 | 0.305±0.025 | 0.152±0.013 | 0.236±0.004 | 0.200±0.041 | 0.128±0.006 | 0.155±0.005 | 0.028±0.038 | 0.021±0.080 | 0.004±0.152 | 0.016±0.104 | 0.043±0.090 | 0.016±0.027 |
| 14027 | 0.317±0.041 | 0.198±0.032 | 0.447±0.035 | 0.240±0.030 | 0.184±0.022 | 0.383±0.010 | 0.049±0.008 | 0.024±0.013 | 0.010±0.032 | 0.021±0.015 | 0.026±0.050 | 0.017±0.027 |
| 14057 | 0.362±0.033 | 0.160±0.036 | 0.426±0.038 | 0.283±0.076 | 0.170±0.090 | 0.326±0.077 | 0.044±0.062 | 0.021±0.153 | 0.011±0.015 | 0.014±0.257 | 0.059±0.036 | 0.017±0.035 |
| 14059 | 0.355±0.045 | 0.048±0.039 | 0.320±0.027 | 0.312±0.055 | 0.039±0.039 | 0.221±0.064 | 0.034±0.081 | 0.015±0.059 | 0.006±0.079 | 0.013±0.129 | 0.041±0.042 | 0.015±0.026 |
| 14055 | 0.435±0.040 | 0.144±0.029 | 0.593±0.029 | 0.385±0.031 | 0.110±0.033 | 0.472±0.051 | 0.059±0.059 | 0.021±0.057 | 0.013±0.072 | 0.018±0.036 | 0.023±0.139 | 0.019±0.010 |
| C103 | 0.485±0.098 | 0.254±0.042 | 0.560±0.046 | 0.376±0.086 | 0.173±0.053 | 0.326±0.088 | 0.043±0.035 | 0.026±0.036 | 0.008±0.307 | 0.021±0.091 | 0.000±0.000 | 0.024±0.011 |
| Mean | 0.362±0.228 | 0.142±0.450 | 0.421±0.258 | 0.302±0.238 | 0.121±0.426 | 0.311±0.285 | 0.043±0.203 | 0.021±0.133 | 0.008±0.338 | 0.019±0.234 | 0.039±0.4857 | 0.019±0.163 |
| Maximum | 0.485 | 0.254 | 0.593 | 0.41 | 0.184 | 0.472 | 0.059 | 0.026 | 0.013 | 0.029 | 0.061 | 0.024 |
| Minimum | 0.208 | 0.046 | 0.236 | 0.2 | 0.039 | 0.155 | 0.028 | 0.015 | 0.004 | 0.013 | 0.000 | 0.015 |
| Variation | 22.78% | 45.01% | 25.84% | 23.78% | 42.65% | 28.51% | 20.33% | 13.29% | 33.81% | 23.59% | 48.57% | 16.26% |
| Sample | T-g | T-m | T-a | T-e |  | To-G | To-D | To-GL |  |  | Total |  |
| ND12 | 0.980±0.037 | 0.820±0.032 | 0.078±0.053 | 0.114±0.079 |  | 0.866±0.032 | 0.953±0.039 | 0.173±0.068 |  |  | 1.992±0.036 |  |
| 14011 | 0.629±0.010 | 0.553±0.016 | 0.063±0.030 | 0.096±0.038 |  | 0.594±0.004 | 0.477±0.004 | 0.270±0.038 |  |  | 1.341±0.008 |  |
| 14022 | 0.917±0.021 | 0.756±0.024 | 0.076±0.022 | 0.080±0.024 |  | 0.753±0.025 | 0.689±0.017 | 0.386±0.012 |  |  | 1.828±0.017 |  |
| 14015 | 0.692±0.015 | 0.484±0.018 | 0.054±0.039 | 0.074±0.078 |  | 0.411±0.003 | 0.549±0.006 | 0.344±0.021 |  |  | 1.305±0.008 |  |
| 14027 | 0.962±0.036 | 0.807±0.018 | 0.082±0.011 | 0.064±0.028 |  | 0.857±0.023 | 0.627±0.031 | 0.432±0.026 |  |  | 1.916±0.026 |  |
| 14057 | 0.948±0.006 | 0.779±0.078 | 0.076±0.070 | 0.089±0.060 |  | 0.780±0.053 | 0.702±0.021 | 0.410±0.033 |  |  | 1.892±0.036 |  |
| 14059 | 0.723±0.036 | 0.571±0.051 | 0.055±0.063 | 0.069±0.046 |  | 0.561±0.040 | 0.714±0.044 | 0.143±0.039 |  |  | 1.418±0.040 |  |
| 14055 | 1.172±0.032 | 0.967±0.041 | 0.092±0.060 | 0.059±0.066 |  | 1.097±0.038 | 0.897±0.036 | 0.297±0.037 |  |  | 2.291±0.037 |  |
| C103 | 1.299±0.053 | 0.875±0.076 | 0.077±0.45 | 0.045±0.045 |  | 0.918±0.060 | 0.925±0.064 | 0.452±0.042 |  |  | 2.295±0.054 |  |
| Mean | 0.925±0.225 | 0.735±0.209 | 0.073±0.165 | 0.077±0.256 |  | 0.760±0.259 | 0.726±0.218 | 0.323±0.325 |  |  | 1.809±0.197 |  |
| Maximum | 1.172 | 0.967 | 0.092 | 0.114 |  | 1.097 | 0.953 | 0.452 |  |  | 2.295 |  |
| Minimum | 0.629 | 0.484 | 0.054 | 0.045 |  | 0.411 | 0.477 | 0.143 |  |  | 1.305 |  |
| Variation | 22.47% | 20.85% | 16.46% | 25.62% |  | 25.91% | 21.81% | 32.45% |  |  | 19.73% |  |

All samples were measured in triplicate. The data are expressed as the means±standard deviation based on dry weight. Abbreviations: DG: daidzin, GLG: glycitin, GEG: genistin, MD: malonyldaidzin, MGL: malonylglycitin, MG: malonylgenistin, AD: acetyldaidzin, AGL: acetylglycitin, AG: acetylgenistin, DE: daidzein, GLE: glycitein, GE: genistein. T-e: aglycone (GE+DE+GLE), T-g: glucoside (GEG+DG+GLG), T-m: malonylglucoside (MG+MD+MGL), T-a: acetylglucoside (AG+AD+AGL). To-G: G-type isoflavonoids (GE+GEG+AG+MG), To-D: D-type isoflavonoids (DE+ DG+ AD+ MD), To-GL: GL-type isoflavonoids (GLE+ GLG+ AGL+ MGL).

**Supplementary content–Table S2.** Isoflavonoids variation (mg/g, D.W.) of soybean varieties under different light treatments in different types of soybean germplasm

| Compound | C103 | | |  | ND12 | | | |
| --- | --- | --- | --- | --- | --- | --- | --- | --- |
|  | Seed | Leaf | |  | Seed | Leaf | |  |
|  |  | CK | Shading |  |  | CK | Shading |  |
| DG | 0.485±0.034b | 0.432±0.006c | 0.439±0.004bc |  | 0.465±0.046bc | 0.473±0.006bc | 0.564±0.013a | |
| GLG | 0.254±0.008a | 0c | 0c |  | 0.046±0.025b | 0c | 0c | |
| GEG | 0.560±0.018bc | 0.273±0.025d | 0.633±0.056b |  | 0.468±0.030c | 0.470±0.002c | 1.177±0.075a | |
| MD | 0.376±0.023a | 0.205±0.006c | 0.210±0.000c |  | 0.410±0.031a | 0.220±0.002c | 0.267±0.007b | |
| MGL | 0.173±0.006a | 0.018±0.000c | 0.018±0.000c |  | 0.044±0.057b | 0.018±0.000c | 0.018±0.000c | |
| MG | 0.326±0.020c | 0.177±0.005e | 0.337±0.017bc |  | 0.366±0.035b | 0.237±0.005d | 0.473±0.014a | |
| AD | 0.043±0.001b | 0c | 0c |  | 0.049±0.051a | 0c | 0c | |
| AGL | 0.026±0.001a | 0c | 0c |  | 0.022±0.044b | 0c | 0c | |
| AG | 0.008±0.002a | 0b | 0b |  | 0.006±0.116a | 0b | 0b | |
| DE | 0.021±0.001b | 0c | 0c |  | 0.029±0.048a | 0c | 0c | |
| GLE | 0b | 0b | 0b |  | 0.061±0.121a | 0b | 0b | |
| GE | 0.024±0.000c | 0.018±0.003c | 0.038±0.005b |  | 0.024±0.026c | 0.062±0.002a | 0.052±0.006a | |
| Total Isoflavone | 2.295±0.088b | 1.123±0.034f | 1.676±0.046d |  | 1.992±0.036c | 1.480±0.013e | 2.551±0.070a | |

All samples were measured in triplicate. The data are expressed as the means±standard deviation based on dry weight. Abbreviations: DG: daidzin, GLG: glycitin, GEG: genistin, MD: malonyldaidzin, MGL: malonylglycitin, MG: malonylgenistin, AD: acetyldaidzin, AGL: acetylglycitin, AG: acetylgenistin, DE: daidzein, GLE: glycitein, GE: genistein. Diﬀerent letters in the same line indicate signiﬁcant (*P*<0.05) diﬀerences between soybean germplasm types with different levels of shade tolerance.

**Supplementary content–Table S3.** Linear ranges and correlation coefficients of the twelve isoflavones in HPLC analysis

| No. | Analyte | Linear range (μg·mL^-1^) | Calibration Equation (y=ax+b) | Correlation coefficients |
| --- | --- | --- | --- | --- |
| Iso-1 | DG | 9.450-189.000 | Y = 2E+07x - 163764 | *R*² = 0.9988 |
| Iso-2 | GLG | 0.087-34.800 | Y = 4E+07x + 30895 | *R*² = 0.9992 |
| Iso-3 | GEG | 3.580-179.000 | Y = 2E+07x + 135544 | *R*² = 0.9998 |
| Iso-4 | MD | 4.000-100.000 | Y = 2E+07x - 81598 | *R*² = 0.9994 |
| Iso-5 | MGL | 0.100-20.000 | Y = 4E+07x - 14566 | *R*² = 0.9991 |
| Iso-6 | AD | 0.013-1.000 | Y = 1E+08x + 2315 | *R*² = 0.9998 |
| Iso-7 | AGL | 0.006-1.000 | Y = 1E+08x + 727.25 | *R*² = 0.9991 |
| Iso-8 | MG | 2.000-100.000 | Y = 3E+07x - 13962 | *R*² = 0.9993 |
| Iso-9 | DE | 0.238-190.000 | Y = 5E+06x + 55083 | *R*² = 0.9990 |
| Iso-10 | AG | 0.050-5.000 | Y = 5E+07x + 2889.6 | *R*² = 0.9997 |
| Iso-11 | GLE | 0.153-15.300 | Y = 9E+07x + 39495 | *R*² = 0.9991 |
| Iso-12 | GE | 0.152-9.250 | Y = 8E+07x + 19736 | *R*² = 0.9997 |


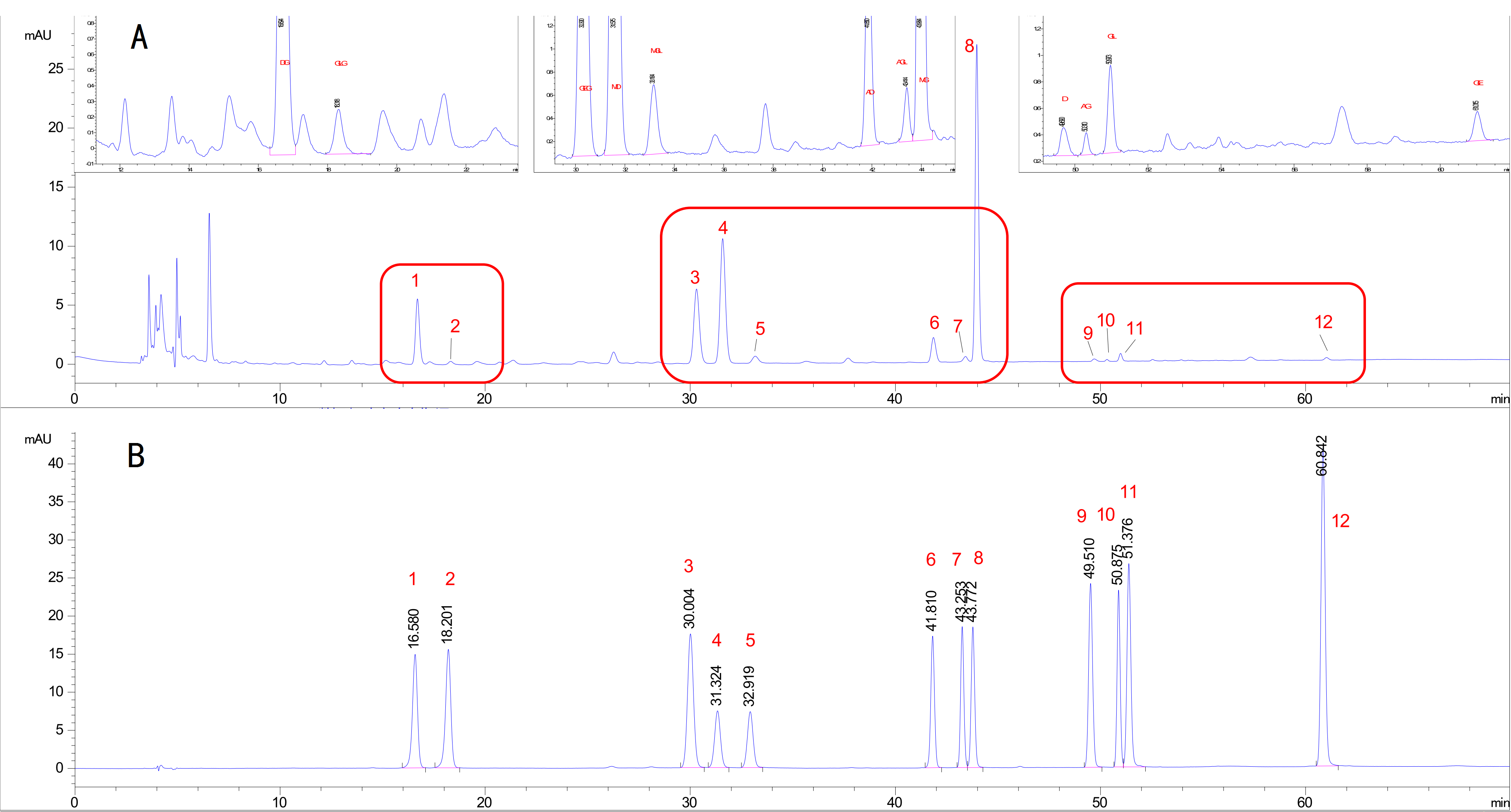


**Supplementary content–Fig. S1.** HPLC chromatograms of soy isoflavone standards and soybean sample. A-Chromatogram of soybean sample; B-Standard chromatogram of soy isoflavone; Peak assignment: peak 1-daidzin (DG); 2-glycitin (GLG); 3-genistin (GEG); 4-malonyldaidzin (MD); 5-malonylglycitin (MGL); 6-acetyldaidzin (AD); 7-acetylglycitin (AGL); 8-malonylgenistin (MG); 9-daidzein (DE); 10-acetylgenistin (AG); 11-glycitein (GLE); 12-genistein (GE)**.**
